# Effects of monensin source on in vitro rumen fermentation characteristics and performance of *Bos indicus* beef bulls offered a high-concentrate diet

**DOI:** 10.1093/tas/txz158

**Published:** 2019-10-01

**Authors:** Daniel A A Teixeira, Bruno I Cappellozza, Juliano R Fernandes, Kaique S Nascimento, Lorena E L M Bonfim, Catarina N Lopes, Jorge A C Ehrhardt, José R Peres, Simon A Harris, José M C Simas, Leo F Richardson

**Affiliations:** 1 Universidade Federal de Goiás, Departamento de Zootecnia, Goiânia, GO, Brazil; 2 Elanco Animal Health, São Paulo, SP, Brazil; 3 Elanco Animal Health, Basingstoke, UK; 4 Elanco Animal Health, Greenfield, IN

**Keywords:** high-concentrate diet, in vitro, monensin, performance, VFA

## Abstract

In Exp. 1, *Brachiaria ruziziensis* (11.1 % CP) was inoculated or not with two sources of monensin, resulting in three treatments: 1) no monensin inoculation (CONT), 2) 20 mg of monensin sodium-A/kg of DM (Elanco Animal Health; MON-A), and 3) 20 mg of monensin sodium-B/kg of DM (Shandong Qilu King-Phar Pharmaceutical Co. Ltd.; MON-B). Three rumen-fistulated Jersey steers were offered a cool-season forage-based diet and were used as the rumen inoculum donors. Volatile fatty acids concentrations were evaluated at 0, 6, 12, 24, 30, and 48 h after treatment inoculation. Overall, acetate and butyrate concentrations were reduced in MON-A vs. CONT (*P* ≤ 0.02), whereas both monensin products reduced Ac:Pr ratio vs. CONT (*P* ≤ 0.01); however, MON-A also (*P* = 0.05) reduced the Ac:Pr ratio vs. MON-B. A treatment × hour interaction was detected for rumen propionate concentration (*P* = 0.01), primarily because MON-A resulted in greater propionate than CONT and MON-B at 24 and 48 h (*P* ≤ 0.03), but no differences were observed between CONT vs. MON-B (*P* ≥ 0.27). In Exp. 2, 240 Nellore bulls (initial BW = 363.2 ± 40.9 kg) were ranked and blocked according to initial BW, and within blocks animals were allotted into pens (*n* = 10 pens/treatment). Pens were randomly assigned into one of three treatments: 1) corn-based diet with no monensin (CONT), 2) CONT plus 28 mg of MON-A/kg of DM, and 3) CONT plus 28 mg of MON-B/kg of DM. The CONT diet was composed of sugarcane bagasse, ground corn, DDGS, urea, and a mineral-vitamin mix. The experimental period lasted 106 d and was divided into a 21-d adaptation period and an 85-d finishing phase. During the adaptation phase, both monensin sources increased (*P* ≤ 0.01) BW change, ADG, and F:G, as well as reduced DMI variation (*P* = 0.02). When the entire experimental period was evaluated, no treatment effects were detected for final BW, DMI, and ADG (*P* ≥ 0.26). Nonetheless, DMI variation was reduced as monensin was included (*P* = 0.01) and only MON-A improved the efficiency by reducing F:G vs. CONT (*P* = 0.05) and biological efficiency vs. MON-B (*P* = 0.05). Additionally, carcass ADG tended (*P* = 0.10) to be greater for MON-A vs. MON-B, whereas no other differences in the carcass characteristics were observed (*P* ≥ 0.53). In summary, the source of monensin inoculated in vitro and offered to Nellore bulls during the feedlot phase significantly affected the energetic efficiency and the performance of the animals.

## INTRODUCTION

In United States, the Food and Drug Administration (FDA) has approved monensin for use in the beef and dairy industry since 1975 and 2004, respectively (Goodrich et al., 1984; FDA, 2004), with the main premise of improving feed efficiency (**FE**) in beef cattle (Duffield et al., 2012), efficiency of milk production in dairy cattle (Duffield et al., 2008), avoiding ruminal disorders, such as bloat and acidosis (McGuffey et al., 2001), as well as coccidiosis prevention in ruminant animals (Foreyt et al., 1986). In beef cattle, Duffield et al. (2012) reported that monensin improves FE by 6.4% in comparison with groups of animals not fed any ionophore, whereas in dairy cattle, the increase in milk production ranged from 0.7 to 1.3 kg/d and the efficiency of production was improved by 2.5% (Duffield et al., 2008).

Although Rumensin (Elanco Animal Health) has been the only monensin source approved in United States, other monensin-containing products have been available in other countries, such as Brazil and Mexico. The differences on cattle performance induced by these sources of monensin, if any, are unknown. Few studies in the literature have used monensin sources other than Rumensin, but reports in which these sources have been used, no positive results in terms of performance were observed (Erasmus et al., 2008). Additionally, to the best of our knowledge, no other study in the literature has compared, under the same environment, different monensin sources in vitro and in vivo. Based on this rationale, we hypothesized that the source of monensin would lead to different responses on in vitro rumen fermentation characteristics and performance of the animals during feedlot. Hence, our objective was to evaluate the effects of monensin sources inoculated in vitro (Exp. 1) and on performance of finishing animals during feedlot and offered a high-concentrate diet (Exp. 2).

## MATERIALS AND METHODS

### Experiment 1: Pilot In Vitro Trial

This experiment was conducted at the Dairyland Laboratories Inc. (Arcadia, WI) from March to June 2017.

A pilot study was conducted, using a single substrate source, to evaluate whether differences between the monensin products would be observed under an in vitro screening. The substrate used was a *Brachiaria ruziziensis* with the inclusion or not of two monensin sources, resulting in three treatments: 1) negative control: no additive inoculation into the forage source (CONT), 2) monensin sodium-A (Elanco Animal Health, Greenfield, IN; MON-A), and 3) monensin sodium-B (Shandong Qilu King-Phar Pharmaceutical Co. Ltd., Jinan, China; MON-B). The monensin-containing products were inoculated under the same dosage (20 mg/kg of DM), whereas four runs were analyzed and within run, samples were analyzed in triplicates.

#### Sampling.

Samples were analyzed in duplicates by wet chemistry procedures for concentrations of CP (method 984.13; AOAC, 2006), NDF (Van Soest et al., 1991; modified for use in an Ankom-200 fiber analyzer, Ankom Technology Corp.), ADF (method 973.18 modified for use in an Ankom-200 fiber analyzer; Ankom Technology Corp., Fairport, NY; AOAC, 2006), and ash (method 942.05; AOAC, 2012). Calculations for TDN used the equation proposed by Owens et al. (2010). The nutritional composition of the forage substrate was 94.3% DM, 11.1% CP, 65.2% NDF, 43.7% ADF, 11.2% ash, and 61.5% TDN.

#### Culture media preparation.

Culture media for all in vitro analyses were composed of calcium chloride dehydrate, manganese chloride tetrahydrate, cobalt chloride tetrahydrate, ferric chloride hexahydrate, and deionized water (DIH_2_O), which was called as a micro solution. The buffer solution contained ammonium bicarbonate, sodium bicarbonate, and DIH_2_O. The macro solution was composed of sodium phosphate, DIH_2_O, and potassium phosphate. The resazurin solution contained resazurin and DIH_2_O, whereas L-cysteine HCl, DIH_2_O, sodium hydroxide, and sodium sulfide were part of the reducing solution. Finally, the incubation buffer contained DIH_2_O, trypticase peptone, and a mixture of buffer, macro, micro, and resazurin solutions.

The incubation buffer with reducing solution was also prepared and adequate time was allowed for the solution to be cooled-off at room temperature while under CO_2_. Appropriate amount of reducing solution was added to the incubation buffer after the solution has been cooled-off.

#### Donor animals and inoculum collection.

Three rumen-fistulated Jersey steers were used as the inoculum source for the present study. The steers were maintained on a cool-season forage-based diet (10.3% CP, 60.7% NDF), without any nutritional additive such as prebiotics, probiotics, enzymes, ionophores, and non-ionophores, in order to avoid any confounding effect with the ionophore being tested herein. The animals were fed twice a day (0700 and 1700 h) and had ad libitum access to the diet. Rumen contents were collected prior to the morning feeding (12–16 h after the evening feeding). Trained personnel collected rumen contents from the three steers by manually taking samples from the fistula and the top layer solids in the rumen were discarded and the rumen contents were stored into appropriate thermos until these were 2/3 full. The same procedure was repeated until three thermoses were obtained for the conduction of the assays. In the lab, the rumen inoculum was placed in a 39°C water bath (water added in the same morning) for warming. The contents were squeezed into three layers of cheesecloth to obtain the liquid phase of the material and pooled into thermoses, whereas the solid part was blended, and the material filtered in cheesecloth sheets to separate the liquid and solid fractions. The cylinder used to store the liquid was kept in a 39°C water bath until further analysis.

#### Laboratorial analysis.

Evaluations for VFA were performed at 0 (immediately prior to inoculation), and approximately at 6, 12, 24, 30, and 48 h post-treatment inoculation. Volatile fatty acids were analyzed as described by Harmon et al. (1985) and from these results, the total VFA concentration and the acetate:propionate (Ac:Pr) ratio were calculated.

### Experiment 2: Performance Trial

This study was conducted at the University Federal de Goiás (UFG; Goiânia, GO, Brazil; 16°40′43″ S, 49°15′14″ W, and 749 m elevation) from April to August 2018. All animals used in the present study were cared for in accordance with acceptable practices and experimental protocols reviewed and approved by the UFG Institutional Animal Care and Use Committee.

#### Animals, housing, and diets.

On day 0 of the study, 240 Nellore bulls (initial BW 363 ± 41 kg) were assigned to 30 pens (soil-surface; 15 × 20 m) in a randomized complete block design according to their initial shrunk BW. Within blocks (*n* = 10), animals were randomly assigned to pens (8 animals/pen) and pens within blocks randomly assigned to one of three treatments: 1) high-concentrate diet without ionophore addition (CONT; *n* = 10), 2) CONT diet plus 28 mg of monensin sodium-A/kg of DM (Elanco Animal Health; MON-A; *n* = 10), and 3) CONT diet plus 28 mg of monensin sodium-B/kg of DM (Shandong Qilu King-Phar Pharmaceutical Co. Ltd.; MON-B; *n* = 10). The basal CONT diet contained sugarcane bagasse, ground corn, dried distillers grains (DDGS; FlexyPro; SJC Bioenergia, Quirinópolis, GO, Brazil), urea, and a mineral-vitamin mix. All animals were vaccinated against foot-and-mouth disease (Aftobov Oleosa; Merial Saúde Animal; Paulínia, SP, Brazil), individually identified with an unique ear tag, received a topical cypermethrin (Cypermil Pour-on; Ourofino Saúde Animal; Cravinhos, SP, Brazil), and were treated for external and internal parasites with albendazole (Ricobendazole 10; Ouro Fino Saúde Animal).

The experimental period lasted 106 d and was divided into an adaptation phase (days 0 to 20) and a finishing phase (days 21 to 105). Cattle were fed once daily (0700 h) and were allowed ad libitum access to feed and fresh water for 106 d. The adaptation phase was performed as a step-up protocol, divided into three periods of 7 d each. The management consisted in reducing the inclusion of sugarcane bagasse by 5% every 7 d, whereas the diet offered during the first and third 7-d period contained (dry matter [DM] basis) 25% and 15% of sugarcane bagasse, respectively. During the finishing phase, the diet was composed of (DM basis) 10% sugarcane bagasse, 75.7% ground corn, 11.7% of DDGS, 0.5% urea, and 2.1% of a mineral-vitamin mix (Ganho Nutrição Animal, Goiânia, GO, Brazil). The nutritional profile of the experimental diets is presented in Table 1.

**Table 1.** Nutritional profile of the experimental diets used during the finishing phase

	*ADAP-1* ^1^		*ADAP-2* ^1^		*ADAP-3* ^1^		*Finishing* ^1^	
Item	CONT	MON	CONT	MON	CONT	MON	CONT	MON
Ingredient, % DM								
Sugarcane bagasse	25.0	25.0	20.0	20.0	15.0	15.0	10.0	10.0
Ground corn	60.7	60.7	65.7	65.7	70.7	70.7	75.7	75.7
Dried distillers grains with solubles	11.7	11.7	11.7	11.7	11.7	11.7	11.7	11.7
Urea	0.5	0.5	0.5	0.5	0.5	0.5	0.5	0.5
Mineral-vitamin mix	2.1	2.1	2.1	2.1	2.1	2.1	2.1	2.1
Monensin, mg/kg	–	28	–	28	–	–	–	28
Nutritional composition, % DM								
Dry matter	68.4	68.4	71.7	71.7	75.4	75.4	79.5	79.5
Crude protein	12.6	12.6	12.9	12.9	13.1	13.1	13.4	13.4
Ether extract	3.3	3.3	3.4	3.4	3.6	3.6	3.7	3.7
Ash	2.8	2.8	2.6	2.6	2.3	2.3	2.1	2.1
Neutral detergent fiber (NDF)	29.8	29.8	26.5	26.5	23.2	23.2	20.0	20.5
Non-fibrous carbohydrate	52.3	52.3	55.5	55.5	58.7	58.7	61.8	61.8
Starch	44.0	44.0	47.5	47.5	51.1	51.1	54.7	54.7
Physically effective NDF	9.5	9.5	8.1	8.1	6.7	6.7	5.3	5.3
Total digestible nutrients	73.7	73.7	75.8	75.8	77.9	77.9	80.1	80.1
Digestible energy, Mcal/kg^1^	3.25	3.25	3.34	3.34	3.44	3.44	3.53	3.53
Metabolizable energy, Mcal/kg^1^	2.66	2.73	2.74	2.80	2.82	2.88	2.90	2.96
Net energy for maintenance, Mcal/kg^1^	1.74	1.79	1.81	1.86	1.88	1.93	1.94	2.00
Net energy for gain, Mcal/kg^1^	1.12	1.16	1.18	1.22	1.24	1.29	1.30	1.35

Experimental diets were offered on a daily basis throughout the experimental period (days 0 to 105). CONT = high-concentrate diet without the addition of monensin; MON = CONT + addition of 28 mg of monensin sodium-A (MON-A; Rumensin-200; Elanco Animal Health) or monensin sodium-B/kg of DM (MON-B; Shandong Qilu King-Phar Pharmaceutical Co. Ltd., Jinan, China).

^1^ADAP-1 = adaptation diet offered from days 0 to 6; ADAP-2 = adaptation diet offered from days 7 to 13; ADAP-3 = adaptation diet offered from days 14 to 20; Finishing period = days 21 to 105.

^2^Calculated according to equations described in NRC (1996).

Throughout the experimental period (days 0 to 105), the ingredients used in the diets were individually weighed into polypropylene feed bags identified with the pen number (1 to 30), transported using wheelbarrows, and dumped into the respective feed bunks (2.85 m linear bunk space, which allowed 0.36 m/bull, one-side only and bunk was located in the middle of each experimental pen). Moreover, the monensin dosage offered to the animals from MON-A and MON-B was based on the previous day total DMI so that both groups were receiving 28 mg of monensin/kg of DM.

#### Sampling.

At the beginning (day 0) and end of the experimental period (day 105), shrunk BW was recorded after 16 h of feed and water withdrawal for average daily gain (ADG) calculation. Throughout the experimental period (days 0 to 105), total DMI was recorded daily by collecting and weighing feed refusals. Additionally, the variation of total daily DMI by the pens (DMI_var) also was calculated for the adaptation phase (days 0 to 20) and the overall feeding period (days 0 to 105). Samples of the offered and nonconsumed diet were collected daily from each pen and dried for 24 h at 105 ± 5°C in forced-air ovens for DM calculation. Additionally, samples of the ingredients used in the diets offered to the animals during adaptation and finishing were collected for further laboratorial analysis. After the experiment, samples of ingredients were thawed, dried in a forced-air oven at 55 ± 5°C for 72 h, and ground using a Wiley mill (Tecnal TE-650; Tecnal Equipamentos Científicos, Piracicaba, São Paulo, Brazil) to pass through a 1-mm screen. Samples were analyzed in duplicate for laboratorial DM (method 930.15; AOAC, 2000), ash (method 942.05; AOAC, 2000), CP (nitrogen × 6.25; method 984.13; AOAC, 2000), ash-corrected NDF using heat-stable α-amylase (A3306; Sigma Chemical. Co., St. Louis, MO) and sodium sulfite omitted (Van Soest et al., 1991), and EE (method 920.39; AOAC, 2000). The NE_m_ and NE_g_ were estimated based on equations described in NRC (1996).

At the end of the study, feed:gain (F:G) and G:F ratios were calculated based on total BW gain and total DMI of the animals. Intermediate full BW measurement was taken on day 20 to evaluate the effects of treatments on the performance of the herd during the adaptation phase (days 0 to 20). All animals were slaughtered on day 106 of the study, following the final BW measurement and a waiting period of 12 h, in a federally inspected commercial packing plant (Frigorífico Fribraz, Cidade Oriental, GO, Brazil). Hot carcasses were separated into two symmetrical sections, weighed to obtain hot carcass weight (HCW), and individually identified. Dressing percent (DP) was calculated by dividing the HCW by final BW on day 105 of the study. Initial DP of the animals was estimated in 50% and then it was calculated the amount of carcass gained by the animals during the experimental period (days 0 to 105). Carcass ADG was calculated by dividing the carcass gain (in kg) and the number of days on feed (105 d). Additionally, biological efficiency (BE) was determined by dividing the total DMI of the pen by 15 kg of carcass produced during the entire experimental period.

#### Statistical analysis.

In Experiment 1, for all the analysis performed herein, the bottle that received the final treatment (substrate ± additive) was considered the experimental unit. All data were analyzed using the PROC MIXED procedure of SAS (Version 9.4; SAS Inst. Inc.; Cary, NC) and the Satterthwaite approximation to determine the denominator df for the test of fixed effects. The model statement contained the effects of substrate, additive, and hour. The specified term for the repeated statement was hour variable, the subject was sample(substrate × additive), and the covariance structure was the compound symmetry, which provided the best fit for these analyses according to the Akaike Information Criterion. Additionally, VFA data obtained at 0 h (immediately before treatment inoculation) were used as covariate. Results are reported as least square means, separated using the PDIFF structure, whereas significance was set at *P* ≤ 0.05 and tendencies were denoted if *P* > 0.05 and *P* ≤ 0.10. Results are reported according to the main effects if no interactions were significant or according to the highest-order interaction detected.

For all the variables analyzed in Experiment 2, pen was considered the experimental unit and all the data were analyzed using the PROC MIXED procedure of SAS (Version 9.4; SAS Inst. Inc.; Cary, NC) and the Satterthwaite approximation to determine the denominator df for the test of fixed effects. For DMI and DMI_Var analysis, the model statement contained the effects of treatment, day, block, and the resulting interactions. Data were analyzed using pen(treatment) as the random variable, given that DMI was recorded from each pen. The specified term for the repeated statement was day, the subject was pen(treatment), and the covariance structure was first-order autoregressive, which provided the best fit for these analyses according to the smallest Akaike Information Criterion. For all the other performance and carcass characteristics data, the model statement contained the effects of treatment, block, and the resulting interaction whereas pen(treatment) and bull(pen) were denoted as random variables. Results are reported as least square means and were separated using the PDIFF structure. For all the data, significance was set at *P* ≤ 0.05 and tendencies were denoted if *P* > 0.05 and *P* ≤ 0.10. Results are reported according to the main effects if no interactions were significant.

## RESULTS AND DISCUSSION

### Experiment 1

The main goal of the present study was to evaluate whether the source of monensin would lead to different rumen fermentation results, primarily VFA. This hypothesis was originated from the fact that several monensin-containing products are available in the market of Brazil and Mexico and the question remains regarding if there are differences on rumen efficacy, including rumen fermentation and digestibility, which in turn, might reflect in different performance results (production efficiency of milk and meat).

Values obtained at 0 h were not significant covariates (*P* ≥ 0.39) and did not differ among treatments for acetate, propionate, butyrate, and the Ac:Pr ratio (*P* ≥ 0.11), but differences were observed on the total VFA (*P* = 0.05; 26.96, 26.72, and 25.99 mM for CONT, MON-A, and MON-B, respectively; SEM = 0.285). During the experiment, treatment effects were observed on rumen acetate (*P* = 0.05), propionate (*P* < 0.01), butyrate (*P* = 0.05), and the Ac:Pr ratio (*P* < 0.001), whereas no differences were detected for total VFA (*P* = 0.70; Table 2). Overall, in vitro inoculation of MON-A yielded a reduced concentration of acetate and butyrate when compared with CONT (*P* ≤ 0.02), whereas no further differences were observed between CONT vs. MON-B (*P* ≥ 0.22) and MON-A vs. MON-B (*P* ≥ 0.12). Conversely, both monensin products resulted in a reduced Ac:Pr ratio compared with CONT (*P* ≤ 0.01), but MON-A also (*P* = 0.05) reduced the Ac:Pr ratio when compared with the MON-B treatment (Table 2).

**Table 2.** In vitro rumen volatile fatty acid (VFA) concentrations of *B. ruziziensis* (11.1% CP; 61.5% TDN) inoculated or not (*n* = 12) with monensin sodium-A (MON-A; Rumensin-200; Elanco Animal Health; *n* = 12) or monensin sodium-B (MON-B; Shandong Qilu King-Phar Pharmaceutical Co. Ltd.; *n* = 12) during Exp. 1

	Treatment				*P*		
Item	CONT	MON-A	MON-B	SEM	Trt	Hour	Trt × Hour
VFA, mM/mol							
Acetate	48.33^b^	46.46^a^	47.34^ab^	0.556	0.05	< 0.0001	0.36
Propionate	15.77^a^	16.44^b^	15.98^a^	0.147	< 0.01	< 0.0001	0.01
Butyrate	7.41^b^	7.23^a^	7.35^ab^	0.057	0.05	< 0.0001	0.27
Total VFA	79.87	79.07	79.77	0.724	0.70	< 0.0001	0.29
Ac:Pr Ratio	3.18^c^	3.00^a^	3.07^b^	0.029	< 0.0001	< 0.0001	0.09

Samples were analyzed in four independent runs and within run, samples were analyzed in triplicates (*n* = 12/treatment). Ionophores (MON-A and MON-B) were added at a steady dose of 20 mg/kg of DM. Within a row, means without a common superscript differ (*P* ≤ 0.05).

**Figure 1.**
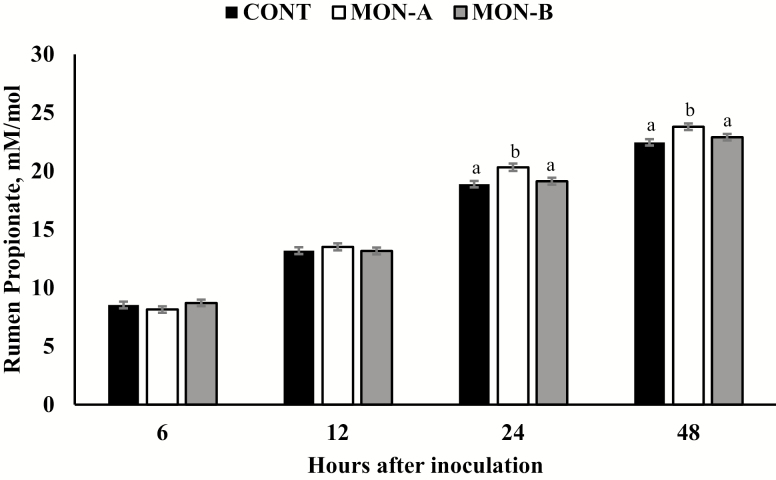
In vitro rumen propionate concentration over the experimental period. *Brachiaria ruziziensis* (11.1% CP; 61.5% TDN) was inoculated or not with monensin-A (MON-A; Rumensin-200; Elanco Animal Health; *n* = 12) or monensin-B (MON-B; Shandong Qilu King-Phar Pharmaceutical Co. Ltd.; *n* = 12). Samples were collected at 0 (immediately before treatment inoculation), 6, 12, 24, and 48 h after treatment inoculation. Results were covariately-adjusted to vales obtained at hour 0. A treatment × hour interaction was detected (*P* = 0.01). Within hour, letters indicate differences between treatments (*P* ≤ 0.03).

Moreover, a treatment × hour interaction was detected for rumen propionate concentration (*P* = 0.01; Figure 1). This interaction was observed primarily because all treatments had a similar propionate concentration at 6 and 12 h after treatment inoculation (*P* > 0.15), whereas MON-A inoculation resulted in greater propionate than CONT and MON-B at 24 and 48 h (*P* ≤ 0.03) and no differences were observed between CONT and MON-B (*P* ≥ 0.27; Figure 1), indicating that one of the monensin sources was not effective in increasing rumen propionate concentrations, as expected and observed after monensin administration in forage-based diets (Dinius et al., 1976; Russell and Strobel, 1989; Packer et al., 2011; Bell et al., 2017).

Ionophores, more specifically monensin, have the ability to change the products of ruminal fermentation and hence, ATP production is potentially affected (Russell and Strobel, 1989). In fact, the relative amounts of VFA produced in the rumen are of particular interest due to their role in metabolic pathways in other organs (Bell et al., 2017). Upon monensin feeding, acetate and butyrate concentrations usually are reduced, whereas rumen propionate concentrations increase (Bergen and Bates, 1984; Spears, 1990). Propionate, a substrate for gluconeogenesis, is the major source of glucose for the ruminant and serves as an H^+^ sink in the rumen, whereas acetate and butyrate are nongluconeogenic and work as H^+^ producers in the rumen (Bell et al., 2017). In other words, propionate production removes H+ ions from the rumen environment and acetate and butyrate production leads to H+ accumulation in the rumen, which may serve as a substrate for the methanogenic bacteria to produce methane (Wolin, 1960). Rumen methane production have two main effects in the rumen and for the animal: 1) removal of H^+^ ions from the rumen, avoiding the reduction in pH and any possible negative effects on the growth and function of cellulolytic bacteria, and 2) energy loss to the ruminant, accounting for up to 12% of the gross energy consumed by the animal (Johnson & Johnson, 1995).

Based on the data above, only one source yielded the results expected and/or desired after monensin addition under the same dosage. Only MON-A (Rumensin-200) inoculation resulted in a greater in vitro rumen propionate and reduced acetate and butyrate concentrations. The exact reason why these differences were observed are unknown at this point, but might be related to the potency of the products (Factor A) and/or to the strain of *Streptomyces cinnamonensins* used for the fermentation of the final product, such as is the case for *Saccharomyces cerevisiae* (Cobos et al., 2010) and the nonionophore antibiotic virginiamycin (Sitta, 2016). Based on these data, the Experiment 02 was designed and conducted in order to evaluate if these differences also would be observed into an in vivo setting with beef animals consuming a high-concentrate diet.

### Experiment 2

The main goal of the present study was to evaluate 1) whether monensin administration into current high-concentrate feedlot diets still provides the expected performance benefits (i.e., feed efficiency), as speculated by Duffield et al. (2012), and 2) whether the source of monensin would lead to different performance results when administered in a high-concentrate feedlot diet. This hypothesis was based on the results obtained in the Exp. 1, in which the source of monensin markedly affected the in vitro fermentation characteristics under a forage-based setting. Additionally, it is imperative to conduct in vivo experiments to support the results of an in vitro study, given the fact that several monensin-containing products are available in Brazil and Mexico market, and the question that remains is whether there are real differences on animal performance when these sources are chosen on a real-life scenario.

For all the variables analyzed and reported herein, no treatment × block interactions were observed (*P* ≥ 0.23). Therefore, all the data will be presented as main effects of treatment and/or any first-order treatment × day interaction for DMI and DMI_var. It is also noteworthy mentioning that no cases of ruminal disorders (i.e., acute acidosis, laminitis, and/or bloat) were observed in the present study, even in animals offered CONT, without any kind of feed additive.

No treatment effects were detected for initial BW (*P* = 0.95), indicating that animals were under the same management prior to the beginning of the study (Table 2). During the adaptation phase (days 0 to 20), no treatment effects were observed (*P* ≥ 0.51) on final BW on day 20, DMI, and DMI as % BW (Table 3). Conversely, treatment effects were observed (*P* ≤ 0.02) on BW change, DMI_var, ADG, F:G, and G:F ratios (Table 3), whereas no further differences were observed due to the source of monensin (*P* ≥ 0.30; Table 3). Body weight evaluation on day 20 of the study was taken when animals had previous ad libitum access to water and feed, whereas the initial BW evaluation was performed by shrinking the animals for 16 h (feed and water). Hence, when a 4% correction factor was used for these initial analyses, the results and treatment effects at the end of the adaptation period were maintained (data not shown) and monensin administration into the diet improved the performance of the herd. This management procedure was adopted in order to avoid impairments on beef herd health and performance, considering that feed and water restriction negatively affect the performance of the beef cattle herd (Marques et al., 2012).

**Table 3.** Performance data of *Bos indicus* bulls receiving a high-concentrate diet containing or not (CONT; *n* = 10) 28 mg of monensin sodium-A (MON-A; Rumensin-200; Elanco Animal Health; *n* = 10) or monensin sodium-B/kg of DM (MON-B; Shandong Qilu King-Phar Pharmaceutical Co. Ltd., Jinan, China; *n* = 10) during the adaptation period (days 0 to 20) of Exp. 2

	Treatment				
Item	CONT	MON-A	MON-B	SEM	*P*
Initial BW, kg	366.5	362.4	360.6	12.76	0.95
Final BW, kg	400.4	405.9	401.5	13.34	0.95
BW Change, kg	33.9^a^	43.5^b^	40.9^b^	2.76	0.01
Dry matter intake, kg	6.71	6.56	6.41	0.169	0.51
Dry matter intake variation, kg	0.483^b^	0.382^a^	0.363^a^	0.0306	0.02
Dry matter intake, % BW	1.75	1.71	1.68	0.060	0.60
Average daily gain, kg/d	1.62^a^	2.09^b^	1.95^b^	0.093	< 0.01
Gain:feed ratio, g/kg	278^a^	360^b^	354^b^	14.2	< 0.001
Feed:gain ratio, kg/kg	3.69^b^	2.81^a^	2.89^a^	0.162	0.001

Diets were offered from days 0 to 20 of the experimental period (adaptation phase). The adaptation phase was divided into three periods of 7 d each and in each period, sugarcane bagasse inclusion decreased from 25% to 15% (DM basis). Within a row, means without a common superscript differ (*P* ≤ 0.05).

Monensin inclusion, independently of the source, in the adaptation diet resulted in a better performance of the herd, by increasing BW change (+8.3 kg), ADG (+0.400 kg/d), G:F (+79 g/kg), and F:G (−0.840 kg/kg) ratios compared with CONT. The improvement in these productive parameters are in the order of 24%, 25%, 28%, and 23% for BW change, ADG, G:F, and F:G, respectively, when compared with the CONT. Our data are in agreement with Duffield et al. (2012) that reported greater feed efficiency when animals are administered monensin vs. unsupplemented monensin cohorts. Additionally, Raun et al. (1976) reported that, compared with unsupplemented monensin cohorts, feed efficiency was improved by 10% and 17% in steers that were supplemented with 11 and 33 mg of MON-A, respectively.

During the overall feeding period (days 0 to 105), no treatment effects were observed (*P* ≥ 0.26) on final BW, BW change, DMI, DMI as % BW, and ADG (Table 4). Conversely, DMI_var, F:G ratio, and BE were affected by the treatments (*P* ≤ 0.05), whereas G:F ratio tended to differ among treatments (*P* = 0.06; Table 4). Similarly to what was observed during the adaptation period, DMI_var was less (*P* = 0.01) for monensin-treated animals compared with CONT cohorts, but similar between the two sources of monensin (*P* = 0.96; Table 4). On the other hand, feeding MON-A during feedlot improved (*P* ≤ 0.02) G:F and F:G ratios when compared with CONT, but no differences were observed between MON-A and MON-B (*P* > 0.13; Table 4). Additionally, MON-B did not positively affect these productive parameters when compared with CONT (*P* > 0.18; Table 4), indicating that the source of monensin offered during the finishing phase of the feedlot plays a key role in regards to the obtained performance of the herd. The present results are in agreement with the results from Exp. 1, in which the source of monensin, when inoculated in vitro affected the rumen fermentation characteristics. More specifically, these authors reported that inoculation with MON-A reduced the molar proportions of acetate and butyrate, as well as increased molar proportions of propionate compared to CONT and MON-B, demonstrating that MON-A resulted in a greater ruminal energetic efficiency (reduced Ac:Pr ratio and methanogenesis) when compared to the other treatments (Richardson et al., 1976). This, in turn, will translate into a greater F:G and G:F ratios and animal performance, as observed in the present experiment and by others (Raun et al., 1976). The improvement on efficiency due to MON-A feeding compared with CONT was in the order of 9.2% and 9.1% for G:F and F:G ratios, respectively, which is greater (6.4 %) than suggested by Duffield et al. (2012). One might speculate that the differences observed between the present study and Duffield et al. (2012) are related to the energy density of the diet. Goodrich et al. (1984) summarized that the optimum diet energy density for monensin addition was 2.9 Mcal of ME/kg of diet DM, which is close to the value reported in Table 1 for the untreated control group, and as the energy density increased above this level, feed efficiency responses might be reduced (Barreras et al., 2013). In the meta-analysis performed by Duffield et al. (2012), the authors noted that, in the last 40 yr, the impact of monensin on feed efficiency was reduced from 8.1% to 3.5%, which may be partially explained by the increases in dietary energy density. However, it is important to mention that most of the studies evaluated by Duffield et al. (2012) were from United States and this rationale may not apply to other geographies (i.e., Brazil), given the differences in diet composition and nutritional profile between U.S. and Brazil feedlot nutritionists recommendations (Millen et al., 2009; Pinto and Millen, 2016; Samuelson et al., 2016).

**Table 4.** Performance data of *Bos indicus* bulls receiving a high-concentrate diet containing or not (CONT; *n* = 10) 28 mg of monensin sodium-A (MON-A; Rumensin-200; Elanco Animal Health; *n* = 10) or monensin sodium-B/kg of DM (MON-B; Shandong Qilu King-Phar Pharmaceutical Co. Ltd., Jinan, China; *n* = 10) during the entire experimental period (days 0 to 105)

	Treatment				
Item	CONT	MON-A	MON-B	SEM	*P*
Final BW, kg	495.8	497.8	488.6	14.38	0.89
BW Change, kg	129.0	136.2	128.0	4.48	0.49
Dry matter intake, kg	7.35	7.13	6.99	0.177	0.37
Dry matter intake variation, kg	0.238^b^	0.201^a^	0.202^a^	0.0093	0.01
Dry matter intake, % BW	2.03	1.97	1.93	0.051	0.37
Average daily gain, kg/d	1.24	1.32	1.23	0.039	0.26
Gain:feed ratio, g/kg	174^a^	190^b^	182^ab^	4.6	0.06
Feed:gain ratio, kg/kg	5.80^b^	5.26^a^	5.52^ab^	0.147	0.05
Biological efficiency, DMI/15 kg carcass	122.9^ab^	114.3^a^	126.1^b^	3.29	0.05

Basal diet contained (DM basis) 10.0% of sugarcane bagasse, 75.7% ground corn, 11.7% dried distillers grains with solubles (FlexyPro; SJC Bioenergia, Quirinópolis, GO, Brazil), 0.5% urea, and 2.1% of a mineral-vitamin mix (Ganho Nutrição Animal; Goiânia, GO, Brazil). Within a row, means without a common superscript differ (*P* ≤ 0.05).

As previously mentioned, monensin supplementation to finishing cattle has been a common practice since its approval in 1975 (Goodrich et al., 1984) and according to recent surveys in Brazil (Pinto and Millen, 2016) and United States (Samuelson et al., 2016). Hence, studies evaluating MON-B supplementation vs. a negative untreated control are even more scarce when compared with MON-A. In one of the few studies available in the literature, Erasmus et al. (2008) reported that supplementation with 15 mg of MON-B/kg of DM did not improve milk production efficiency when compared with a nonsupplemented group of lactating dairy cows. The reason why MON-B did not improve the production efficiency herein, in Exp. 1, and in Erasmus et al. (2008) is unknown at this point, but might be related to the potency of the monensin sodium-containing products (Factor A) and/or to the strain of *Streptomyces cinnamonensins* used for the fermentation of the final product, such as is the case for the strains of *Saccharomyces cerevisiae* (Cobos et al., 2010) and the nonionophore antibiotic virginiamycin (Sitta, 2016).

Another important factor to be considered when evaluating the overall profitability of a feedlot operation is the biological efficiency, given that this trait represents how much feed was required for the animal (total DMI) in order to gain 15 kg of carcass. As aforementioned and reported in Table 3, MON-A supplementation reduced the amount of feed required to gain 15 kg carcass gain when compared with MON-B (*P* = 0.02; + 10.3% improvement) and tended to reduce this number when compared with CONT (*P* = 0.08; + 7.3% improvement), whereas no further differences were observed between CONT and MON-B (*P* = 0.49). These results are in agreement with the improved efficiency parameters reported herein (Table 4), in the Exp. 1, and by others when feeding MON-A to beef cattle (Ribeiro et al., 2015).

This study was designed and tested by power (Faul et al., 2007; G*Power Statistical Software; Heinrich Heine, Universität Düsseldorf, Germany) to detect statistical differences on F:G and G:F ratios between MON-A and CONT, whereas no assessment was performed between MON-A vs. MON-B and MON-B vs. CONT, given the lack of a database and literature studies to perform such analyses. The same rationale was applied to DMI, given that several reports in the literature reported that when monensin was added into a high-concentrate diet, DMI reduction ranged from 5.0% to 7.5% when compared with a nonsupplemented group (Goodrich et al., 1984; Schelling et al., 1984; Russell and Strobel, 1989; Wood et al., 2016). In fact, the numerical reduction in DMI observed during the entire feeding period (days 0–105) by feeding monensin ranged from 3.0% to 5.0%, being in line with Duffield et al. (2012). In agreement with our results, Felix et al. (2012) did not report a decrease in DMI when monensin was added in high-concentrate diets at doses that ranged from 0 to 44 mg/kg of DM. Moreover, Lemos et al. (2016) also reported no differences in feedlot DMI when *B. indicus* cattle were fed a no-roughage finishing diet for 101 d containing MON-A and/or other growth promoting molecules. In the present study, total DMI was less than expected for the CONT (projected mean DMI = 2.30% BW vs. observed mean DMI = 2.03% BW), indicating that other factor(s), such as environmental and dietary, might have prevented a greater DMI from all treatment groups.

Nonetheless, regardless of source, DMI_var was reduced due to monensin feeding, indicating a smaller DMI fluctuation compared to CONT cohorts. Gibb et al. (2001) also reported a reduced DMI_var in heifers supplemented with MON-A vs. salinomycin. Considering that the costs associated with feedstuffs are the greatest factor influencing the profitability of a commercial beef cattle operation, accounting for over 63% of the variation in total annual costs (Miller et al., 2001), the reduced DMI fluctuation observed due to monensin supplementation will improve the predictability of feed intake by the feedlot animals, resulting in reduced amount of feed wasted in the bunk, positively affecting the profitability of the operation, and finally, likely preventing the occurrence of digestive disorders. As reported by others, a greater variation in total DMI is commonly associated with low ruminal pH of cattle fed high-grain diets (Owens et al., 1998; Krehbiel, 2014).

In general, the improvement in performance after monensin feeding is mostly a result of the reduced DMI, a maintenance on ADG, and a subsequent less FE (F:G and/or G:F ratios; Duffield et al., 2012). Spears (1990) reported that ionophores, including monensin, usually do not affect starch digestibility and, in fact, monensin often reduces ruminal starch digestibility, while increasing the amount of starch digested in the intestine. In agreement, McCarthy et al. (2015) also reported that monensin did not affect starch digestibility in dairy cows during the periparturient period. Corn and starch were the primary feedstuff and nutrient found in the diets of the present study (70.7% corn; Table 1), respectively. Additionally, any improvement on performance due to NDF digestibility would be scarce and unexpected, given the increased passage rate often observed in high-concentrate diets, which would cause a reduction on NDF digestibility, and also the significative proportion (as %) of indigestible NDF and lignin found in the sugarcane bagasse (approximately 42% and 21% on a DM basis, respectively; Masarin et al., 2011; Almeida et al., 2018).

No treatment effects were observed on any of the carcass characteristics parameters evaluated herein (*P* ≥ 0.24; Table 5). The only exception is that MON-A supplementation tended (*P* = 0.10) to result in a greater ADG carcass when compared with MON-B (Table 5). The tendency observed between MON-A and MON-B agrees with the previous performance data and also the results from Exp. 1, in which the source of monensin affected in vitro energetic efficiency by alterations on the Ac:Pr ratio. As reported by previous authors (Montgomery et al., 2003; Barreras et al., 2013; Lemos et al., 2016), monensin supplementation did not benefit carcass characteristics when compared with CONT. Beerman (1995) concluded that the effects of ionophores on carcass composition traits are too small to be of economic and productive significance.

**Table 5.** Carcass characteristics data of *Bos indicus* bulls receiving a high-concentrate diet containing or not (CONT; *n* = 10) 28 mg of monensin sodium-A (MON-A; Rumensin-200; Elanco Animal Health; *n* = 10) or monensin sodium-B/kg of DM (MON-B; Shandong Qilu King-Phar Pharmaceutical Co. Ltd., Jinan, China; *n* = 10) during the entire experimental period (days 0 to 105) during Exp. 2

	Treatment				
Item	CONT	MON-A	MON-B	SEM	*P*
Initial carcass Wt., kg	183.2	181.2	180.3	6.38	0.95
Dressing percent, %	55.8	55.3	55.5	0.30	0.54
Hot carcass weight, kg	276.6	275.3	270.4	2.76	0.83
Carcass average daily gain, kg/d	0.898	0.911	0.859	0.0222	0.24
Carcass gain, kg	93.4	94.1	90.2	2.41	0.53

Basal diet contained (DM basis) 10.0% of sugarcane bagasse, 75.7% ground corn, 11.7% dried distillers grains with soluble (FlexyPro; SJC Bioenergia, Quirinópolis, GO, Brazil), 0.5% urea, and 2.1% of a mineral-vitamin mix (Ganho Nutrição Animal; Goiânia, GO, Brazil).

## CONCLUSIONS

In summary, ruminal fermentation characteristics were significantly affected by the source of monensin, in a manner that MON-A improved the energetic efficiency (Ac:Pr) by reducing concentrations of acetate and butyrate and increasing propionate concentrations compared with negative control and MON-B. Additionally, supplementation of *B. indicus* Nellore bulls with monensin during the adaptation phase improved the performance of the herd, independently of the source used. On the other hand, during the entire feeding period (105 d), only MON-A improved feed efficiency vs. CONT, but also improved the biological efficiency vs. MON-B, demonstrating its effectiveness in a current feedlot diet. Therefore, the source of monensin affects the productive responses of *B. indicus* Nellore bulls receiving a high-concentrate diet during the feedlot period.

*Conflict of interest statement*. None declared.
